# 3D Hybrid Scaffolds Based on PEDOT:PSS/MWCNT Composites

**DOI:** 10.3389/fchem.2019.00363

**Published:** 2019-05-21

**Authors:** Akhila K. Jayaram, Charalampos Pitsalidis, Ellasia Tan, Chrysanthi-Maria Moysidou, Michael F. L. De Volder, Ji-Seon Kim, Roisin M. Owens

**Affiliations:** ^1^Department of Chemical Engineering and Biotechnology, University of Cambridge, Cambridge, United Kingdom; ^2^Department of Physics and Centre for Plastic Electronics, Imperial College London, London, United Kingdom; ^3^Department of Engineering, University of Cambridge, Cambridge, United Kingdom

**Keywords:** carbon nanotubes, conducting scaffolds, porous, PEDOT:PSS, electrode

## Abstract

Conducting polymer scaffolds combine the soft-porous structures of scaffolds with the electrical properties of conducting polymers. In most cases, such functional systems are developed by combining an insulating scaffold matrix with electrically conducting materials in a 3D hybrid network. However, issues arising from the poor electronic properties of such hybrid systems, hinder their application in many areas. This work reports on the design of a 3D electroactive scaffold, which is free of an insulating matrix. These 3D polymer constructs comprise of a water soluble conducting polymer (PEDOT:PSS) and multi-walled carbon nanotubes (MWCNTs). The insertion of the MWCNTs in the 3D polymer matrix directly contributes to the electron transport efficiency, resulting in a 7-fold decrease in resistivity values. The distribution of CNTs, as characterized by SEM and Raman spectroscopy, further define the micro- and nano-structural topography while providing active sites for protein attachment, thereby rendering the system suitable for biological/sensing applications. The resulting scaffolds, combine high porosity, mechanical stability and excellent conducting properties, thus can be suitable for a variety of applications ranging from tissue engineering and biomedical devices to (bio-) energy storage.

## Introduction

Conducting polymer (CP) scaffolds belong to a novel class of scaffold materials that combine the softness of the polymer scaffolds and the electrical properties of conducting polymers (Lee, 2013; Wan et al., 2015; Zhou et al., 2018). Their unique set of features, including electrical conductivity, compatibility with tissue and a 3D porous structure that can take any desired form and shape, is reminiscent of their organic nature (Arash et al., 2014; Balint et al., 2014; Wang et al., 2014; Guex et al., 2017). While traditional inorganic materials such as silicon offer room for processing or further modification/functionalization, they are limited by inefficient biological coupling due to the formation of oxide layers upon interfacing with electrolytes (Rivnay et al., 2016). Therefore, CP scaffolds can offer viable alternatives to existing biomaterials for a range of applications, wherein electrical conductivity can be necessary for medical applications such as simulating/recording tissues (Kim et al., 2018), as well as for 3D tissue engineering (Guo and Ma, 2018).

Advances in materials science and tissue engineering as well as manufacturing techniques have enabled the advent of smart, multifunctional scaffolds with tailored architectures able to promote cell alignment and adhesion, release biomolecules on demand, and promote cell growth and differentiation in response to electrical stimulation (Ghasemi-Mobarakeh et al., 2011). Such conducting scaffolds or hybrid structures are typically formed by incorporating an electrically conducting material in insulating hydrophilic networks. Until now, the incorporation of CPs in a prefabricated insulating scaffold has dominated the field of electrically conducting scaffolds (Hardy et al., 2013; Zubair et al., 2017). However, the synthesis of single-component CP scaffolds offers the possibility of improved electrical properties while decreasing the complexity of the system (Wan et al., 2015). Single component CP scaffolds can be formed by either self-assembly of the conjugated polymeric chains or by modifying the CP with water-soluble and chemically crosslinkable moieties. One recent example of a single-component CP scaffold is based on poly(3,4-ethylene dioxythiophene (PEDOT) doped with poly(styrene sulfonate) (PSS) where the latter acts as counter ion for hole doping the CP while allowing it to be water dispersible. Such 3D structures made from PEDOT:PSS have been realized using freeze drying, with high versatility in forms and shapes as well as in the structural and functional properties (Wan et al., 2015; Guex et al., 2017; Iandolo et al., 2018). Recent studies further reported on how the mechanical, electrical and structural properties of the resulting PEDOT:PSS scaffolds could be modified by mixing the CP aqueous dispersion with other materials to meet different application requirements (Inal et al., 2017; Del Agua et al., 2018). Additionally, in our latest work we showed that single walled carbon nanotubes (SWCNTs) addition resulted in a substantial increase in the PEDOT:PSS scaffold conductivity and device performance (Pitsalidis et al., 2018a).

CNTs are 1D materials that demonstrate ballistic transport of electrons under certain conditions (Saito et al., 1998). Resistivity as low as 10^6^ Ω^−1^ cm^−1^ was reported in single walled carbon nanotubes (SWCNTs) (Purewal et al., 2007), while multi-walled carbon nanotubes (MWCNTs) have shown resistivity of 5 × 10^6^ Ω^−1^ cm^−1^ (Schönenberger et al., 1999); the difference being attributed to the change in respective diameters (Lekawa-Raus et al., 2014). The increased length of carbon nanotubes can promote conduction, provided it is within the length scale of the ballistic regime (Sundqvist et al., 2008) and that the CNTs form a good percolated network. One of the most exciting directions of CNT-based materials and structures is the use of CNT/polymer composites due to their enhanced mechanical (high strength and durability upon formation of a percolated network) and electrical properties (Ajayan et al., 2000; Behabtu et al., 2013; Lin et al., 2013; Arash et al., 2014). Indeed, combining CNTs with a conducting polymer greatly improves the charge transport efficiency while renders the resulting structure or film sensitive to chemical and/or environmental changes (Gou et al., 2015). Interestingly, the incorporation of water-soluble conjugated polymers/CNTs mixtures are of great interest for electrochemical devices and biosensors as they can enhance signal transduction and the range of detection (Gao et al., 2003; Xu et al., 2006).

The present study builds on our previous work (Pitsalidis et al., 2018a), aiming to systematically investigate the effect of MWCNTs addition on the PEDOT:PSS scaffold properties. As such we describe the fabrication and characterization of 3D conducting polymer scaffolds based on oxidized high-aspect ratio MWCNTs and PEDOT:PSS mixtures. The resulting 3D electrodes exhibit nanostructured porous morphology, substantially lower impedance compared to pristine scaffolds and excellent cytocompatibility. Furthermore, we demonstrate the biofunctionalization capability of our system using a bioactive peptide [poly-L-lysine (PLL)]. We believe that such 3D hybrid electrodes will open new directions for bio-interfacing and tissue engineering applications.

## Materials and Methods

### Preparation of the PEDOT:PSS/MWCNT Scaffolds

**Figure 1:**
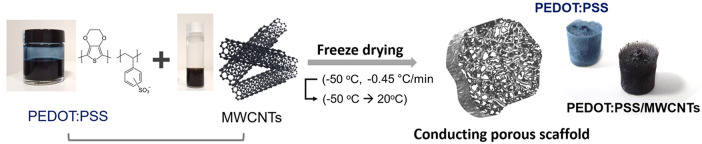
Schematic diagram and images showing the components and the process used for the fabrication of the 3D conducting scaffolds. The photos show free-standing conducting scaffolds based on PEDOT:PSS and PEDOT:PSS/MWCNTs.

The hybrid materials were prepared by mixing PEDOT: PSS and MWCNT solutions in various ratios. MWCNTs from Microphase (LLCNT, 300 μm in length) or Nanocyl (NC7000, 1.5 μm in length) were oxidized using nitric acid (HNO_3_) in a microwave reactor (Anton Paar Multiwave Pro). PEDOT: PSS solution (Clevios PH-1000, Heraeus) of 1.25 wt% was mixed with 0.5 wt% 4-dodecylbenzenesulfonic acid (DBSA, Sigma Aldrich) and 3 wt% 3-glycidoxypropyltrimethoxysilane (GOPS, Sigma Aldrich). GOPS acted as a crosslinker while DBSA was shown to improve conductivity in previous studies (Inal et al., 2017). Subsequently, the above solution was mixed with MWCNTs through sonication for 10 min in the following MWCNT:(PEDOT:PSS) ratios: 1:1 and 2:3. The resultant solution was pipetted into a 96-well plate (Eppendorf) with a volume of approximately 250 μL per well. In order to enable electrical measurements, small strips of gold-plated polyimide (Kapton®, DuPont) were inserted in some of the wells prior to pipetting of solution. The well plate was placed in a freeze dryer (VirTis Advantage Plus) and frozen from 5 to −50°C at a rate of −0.45°C/min. The drying phase involved an initial temperature ramp to −45°C and a subsequent ramp to 20°C. The scaffolds were brought to room temperature after the heat-treatment stage. Prior to characterization, the samples were heated at 70°C on a hotplate for at least 3 h in order to promote the crosslinking of the PEDOT:PSS based scaffolds. A summary of the process and the resulting scaffold structures are shown in Figure 1.

### Characterization of MWCNTs With Raman Spectroscopy

All samples were measured as prepared using Raman spectroscopy acquired from Renishaw inVia Raman Microscope in a backscattering configuration. All measurements were performed with samples placed inside a Linkam THMS600 stage under continuous nitrogen purging. The calibration of the filter and grating were performed using a Si reference peak at 520 cm^−1^. A HeNe laser was used to produce the excitation source at 633 nm and 10% of 12 mW laser intensity under 50x magnification. The Raster 2D mapping was measured at each 10 μm step within filled square area of 40 and 60 μm across both axis.

### Optical Characterization of PEDOT:PSS/MWCNT Scaffolds

The microstructure and surface morphology of the scaffolds were analyzed using Scanning Electron Microscopy (SEM, Helios Nanolab DualBeam, FEI). The samples were removed from the well plate and mounted on an aluminum stub with carbon conductive tape. The beam voltage used was 5 kV and the beam current was maintained at 50 pA.

### Swelling Studies of PEDOT:PSS/MWCNT Scaffolds

The measurement of liquid uptake, or swelling, was performed on the scaffolds by immersing them in Phosphate Buffered Saline (PBS, Sigma Aldrich, pH = 7.4) solution for 2, 4, 6, and 24 h, respectively. The scaffolds were weighed prior to immersion to measure the dry weight (W_d_). Post immersion, they were dried thoroughly with tissue paper and weighed again to obtain the wet weight (W_w_). The following equation was used to calculate the swelling (*L*) of the scaffolds (Shahini et al., 2014; Kucinska-Lipka et al., 2017):

(1) $$\begin{array}{r} L=\;\frac{Ww-Wd\;}{Wd}\;\times 100 \end{array}$$

Two slices and four measurements per scaffold type were used for calculations to account for variability. The mean of these four measurements was taken as the swelling index of the scaffold.

### Pore Size Distribution of PEDOT:PSS/MWCNT Scaffolds

The pore size distribution of the scaffolds was measured using image analysis of SEM micrographs (Park et al., 2002). Fifty pores per sample were manually selected at random and their diameters were measured using *ImageJ*. Further graphical analysis was performed using Origin. Box plots were plotted to enable visualization of differences among samples.

### Biofunctionalization of PEDOT:PSS/MWCNT Scaffolds

Based on previous work of our group (Pappa et al., 2017), we functionalized the scaffolds with poly-L-lysine (PLL). Briefly, samples were fully immersed in a 1 mg/mL fluorescein isothiocyanate (FITC)-labeled PLL (MW: 15–30 kDa, Sigma-Aldrich) solution in Phosphate Buffered Saline (PBS, Sigma Aldrich) and kept at room temperature, in dark overnight. The next day, the scaffolds were immersed in a 1M NaCl solution and sonicated for 1 min to remove the excess of PLL-FITC. Both unwashed and washed samples were mounted on glass bottom microscopy dishes (MatTek Corporation) and FITC fluorescence was detected upon excitation at 488 nm with a confocal microscope (Axio Observer Z1, Carl Zeiss MicroImaging GmbH).

### Electrical Characterization of PEDOT:PSS/MWCNT Scaffolds

The impedance of the electrodes was evaluated using a two-electrode system configuration. The conducting scaffold was designated as the working electrode through the attachment of a gold-plated Kapton® strip while a reticulated (vitreous) glassy carbon was used as the counter electrode, with PBS acting as the electrolyte. AC voltages of frequencies ranging from 0.1 Hz to 10^5^ Hz were applied and the response was measured using an impedance analyzer (Metrohm Autolab).

### 3D Cell Culture Experiments

Telomerase Immortalized Fibroblasts (TIFs), labeled with Red Fluorescent Protein (RFP—TIF LifeAct) were cultured in Advanced DMEM (Gibco, Life technologies) supplemented with 20% Fetal Bovine Serum (FBS, Sigma Aldrich), 1% Glutamine (Gibco, Life technologies), 2% HEPES (Gibco, Life technologies), 0.5% penicillin-streptomycin (10,000 U/ml, Gibco, Life technologies) and 0.1% Gentamycin (Sigma Aldrich). The day before seeding, scaffolds were fully hydrated and kept at 4°C overnight and the next day, they were sterilized with 70% ethanol and then immersed in complete growth medium for 2 h, to allow for protein adhesion. After washing the scaffolds with fresh medium, 100 μL of 2 × 10^6^ cells in suspension were seeded on top of each sample and cells were incubated for 1.5 h. Then fresh medium was added to maintain the cell culture. Two days later, cells in the scaffolds were fixed with 4% paraformaldehyde (PFA) for 15 min and washed extensively with PBS. Then the samples were observed under a confocal microscope (Axio Observer Z1, Carl Zeiss MicroImaging GmbH) to check cell adhesion and proliferation in the porous network of the scaffolds.

## Results and Discussion

### Morphology and Structure

**Figure 2:**
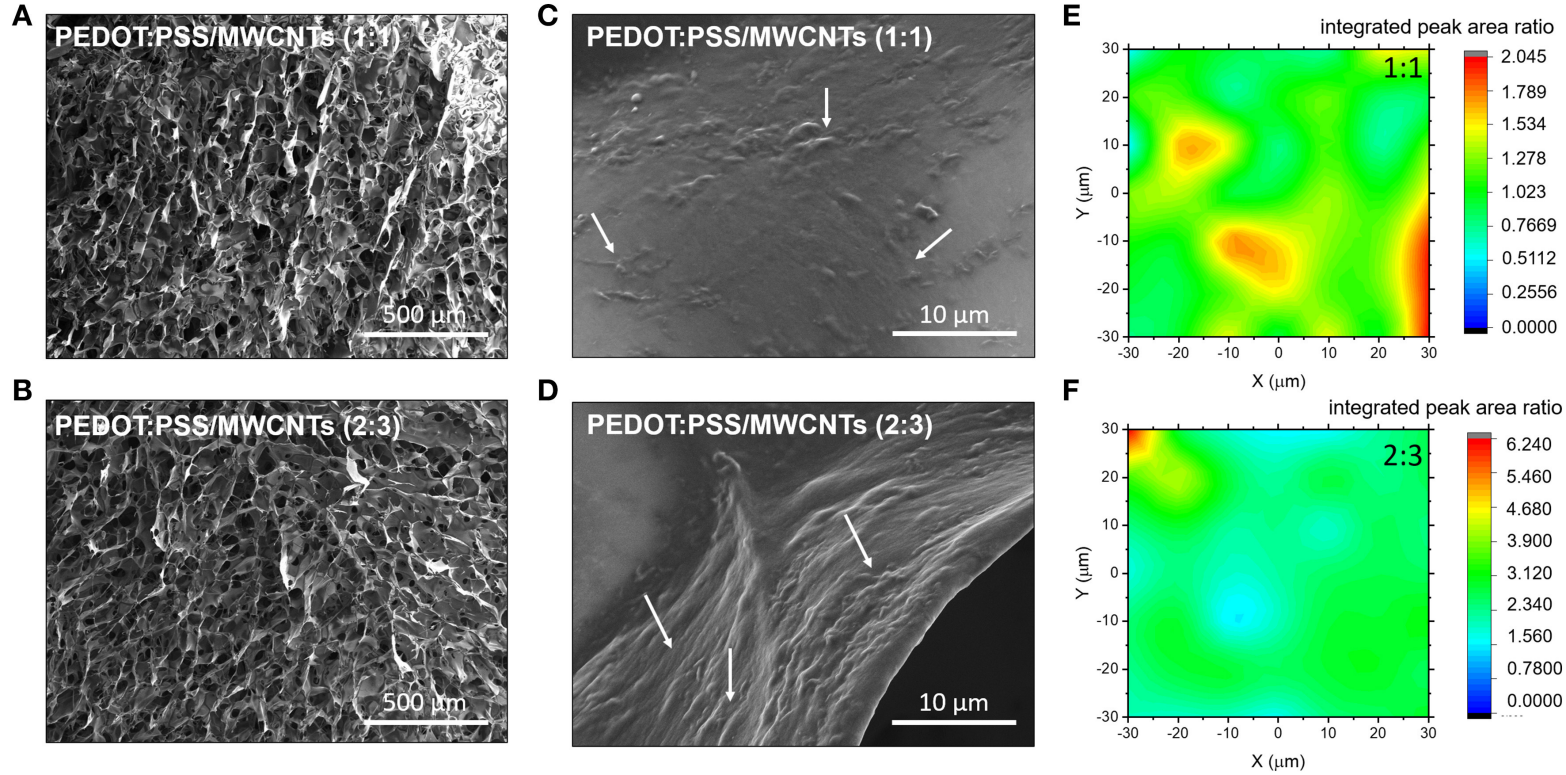
SEM Images of the with PEDOT:PSS/MWCNT scaffold in **(A)** 1:1 ratio and **(B)** 2:3 ratio. White arrows at the corresponding magnified images **(C,D)** highlight the presence of CNT domains. Normalized 2D Raman maps showing integrated peak area ratio of the D-band signal arising from MWCNT to PEDOT:PSS for the different ratio; **(E)** 1:1, **(F)** 2:3.

Visual observations of pristine PEDOT:PSS scaffolds and those with MWCNTs (Figure 1) show a clear color difference, preliminarily indicating the presence of MWCNTs in the resulting structure. The color of the former is bluish, while the latter dispersion is distinctly black, consistent with previous reports showing that CNTs act as black absorbers (Yang et al., 2008). The good dispersibility of oxidized MWCNTs in water facilitates their incorporation within the polymeric matrix. The resulting free-standing scaffolds showed extensive pore interconnectivity and excellent structural integrity both in dry state as well as in wet medium (i.e., PBS). The macroscopic fragility and elasticity of the scaffolds were investigated (2:3 volume ratio) in a qualitative manner by performing compression tests and estimating the response of the material to a unidirectional compressive load (Figure S1). The scaffolds were found to recover their initial shape (in ~10 s) after exposing them to a compression of about 60%, indicative of their good elastic properties and mechanical durability. Figures 2A,B show that the macroscopic porosity of the scaffolds does not change appreciably upon increasing the fraction of MWCNTs in the mixture, within the concentration range tested in this paper. However, the hybrid scaffolds exhibited decreased uniformity when compared to pristine PEDOT:PSS scaffolds, as can be seen in Figure S2. Additionally, the relatively smooth topography observed at the pores of the pristine PEDOT:PSS scaffolds is replaced by a nanostructured topography which is associated with the presence of MWCNTs, as observed in Figures 2C,D. Increasing their fraction (2:3) resulted in a more extensive network of MWCNTs covering the pore surface. The presence of such domains could be linked to phase separation phenomena between PEDOT:PSS and MWCNTs during the freeze drying process.

The elemental composition around these domains was further investigated using Raman spectroscopy. The integrated peak area ratios between the D-band signal from the MWCNT and the symmetric vibrational mode of the PEDOT:PSS C_α_ = C_β_ (1,420 cm-^−1^) were used to construct Raman maps showing MWCNT chemical homogeneity. The domains appear clustered for the hybrid material with lower concentration of MWCNTs, as shown in Figure 2E. In contrast, an even coverage was observed in the material with higher concentration of MWCNTs, as seen in Figure 2F. The presence of nanoscale roughness due to the MWCNT domains is of particular significance to biological applications as cells prefer moderately rough surfaces (Gentile et al., 2010; Zhou et al., 2018). Moreover, such domains can participate in the localization of functionalization moieties. It should be noted that the PEDOT:PSS Raman peaks are strongly influenced by changes in the electrical properties of PEDOT:PSS such as doping level (Garreau et al., 1999). In particular, the principal peak related to the C_α_ = C_β_ symmetric vibrational mode will change with respect to the full width half maximum, peak position and relative intensity ratios to other PEDOT:PSS peaks. Such changes are associated with the structural transformation induced by altering the doping level of PEDOT:PSS. These Raman spectral features are not seen in the PEDOT:PSS/MWCNT hybrid scaffolds (Figure S3), which indicates that the intrinsic doping level of PEDOT:PSS maintained constant between different composition ratios. Thus, suggesting that the enhanced electrical properties are not due to the MWCNTs acting as dopants but alternatively contributing to the conductive pathways with an even coverage in the 3D scaffold.

### Pore Size Distributions

**Figure 3:**
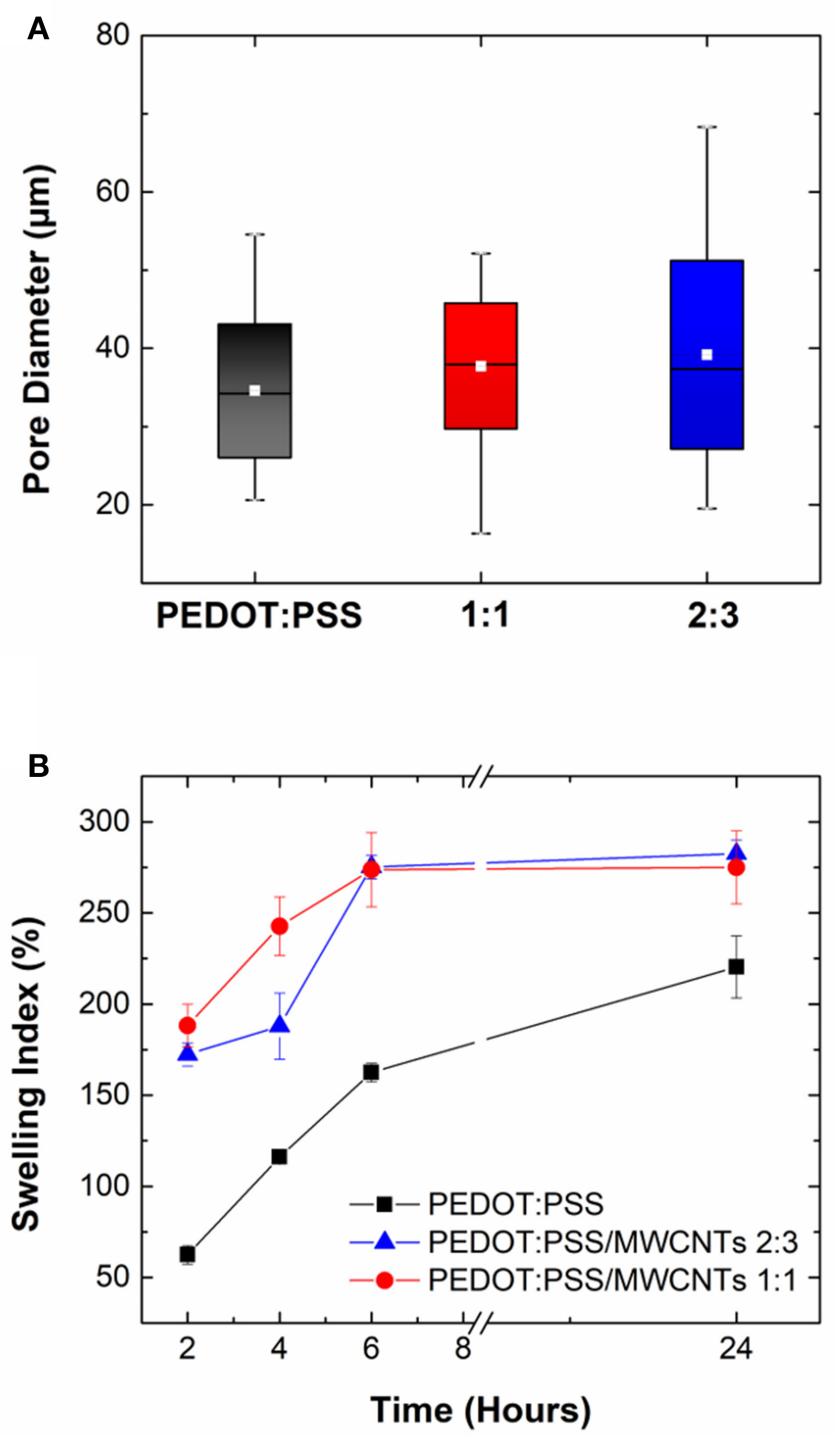
**(A)** Box plot highlighting variation in pore size distribution of PEDOT:PSS/MWCNT scaffold in 1:1 ratio (red), 2:3 ratio (blue) compared to pristine PEDOT:PSS scaffold (black). Colored boxes denote the range where 25–75% of the values in the dataset lie, white line refers to the mean pore diameter and whiskers indicate the maximum and minimum values, respectively. **(B)** Swelling index (%) of PEDOT:PSS/MWCNT scaffold in 1:1 ratio (red) and 2:3 ratio (blue) in comparison with pristine PEDOT:PSS scaffold (black) at *t* = 2, 4, 6, and 24 h. Error bars indicate variability between measurements.

Measurements obtained through image analysis showed that pores in pristine PEDOT:PSS scaffolds had diameters ranging from 20 to 54 μm while PEDOT:PSS/MWCNT scaffolds displayed ranges of 16–52 μm (1:1 ratio) and 19–68 μm (2:3 ratio), respectively. The box plots in Figure 3A indicate that the pristine scaffold has a comparable mean pore diameter (34 μm) to those of the two composites (37 μm, 39 μm). The interquartile range which refers to the range where 25–75% of diameters lie (denoted by the shaded boxes) also follows a similar trend. Hence, the pore diameters obtained are of a sufficient diameter to promote cell growth (Karageorgiou and Kaplan, 2005; Li et al., 2013) which is further corroborated by cytocompatibility studies detailed in 3.6.

### Swelling Capacity

The swelling capacity of PEDOT:PSS/MWCNT scaffolds is seen to be higher than that of pristine PEDOT:PSS scaffolds at all time points, as shown in Figure 3B. However, there is no significant difference between the two PEDOT:PSS/MWCNT scaffolds. Following incubation for 2 h, the former were seen to have a liquid uptake 3 times (172–188%) that of the latter (62%). The trend of higher liquid uptake continued at longer timescales (*t* = 24 h), although the difference among the samples was now lowered (275–282.5% for CNT scaffolds vs. 220% for pristine scaffolds). The differences can be attributed to the lower relative content of PSS in the PEDOT:PSS/MWCNT scaffolds, as well as to the increase in surface area arising from the microstructure imparted by the CNTs. The swelling capacity is of significant importance for the use of these scaffolds in tissue engineering applications as the ability to retain water promotes cell proliferation and perfusion of nutrients (Zhu and Marchant, 2011; Slaughter et al., 2013).

### Electrical Properties

The macroscopic conductivity of the MWCNTs-based scaffolds in their dry form was assessed by measuring the resistance between two contact points of the scaffolds (Figure S4). The incorporation of MWCNTs was found to have a pronounced effect on the conductivity of the scaffolds, as expected. The measured electrical resistance was approximately 7 times lower compared to the pristine PEDOT:PSS samples, while only slight variations were observed between the two different MWCNTs ratios.

**Figure 4:**
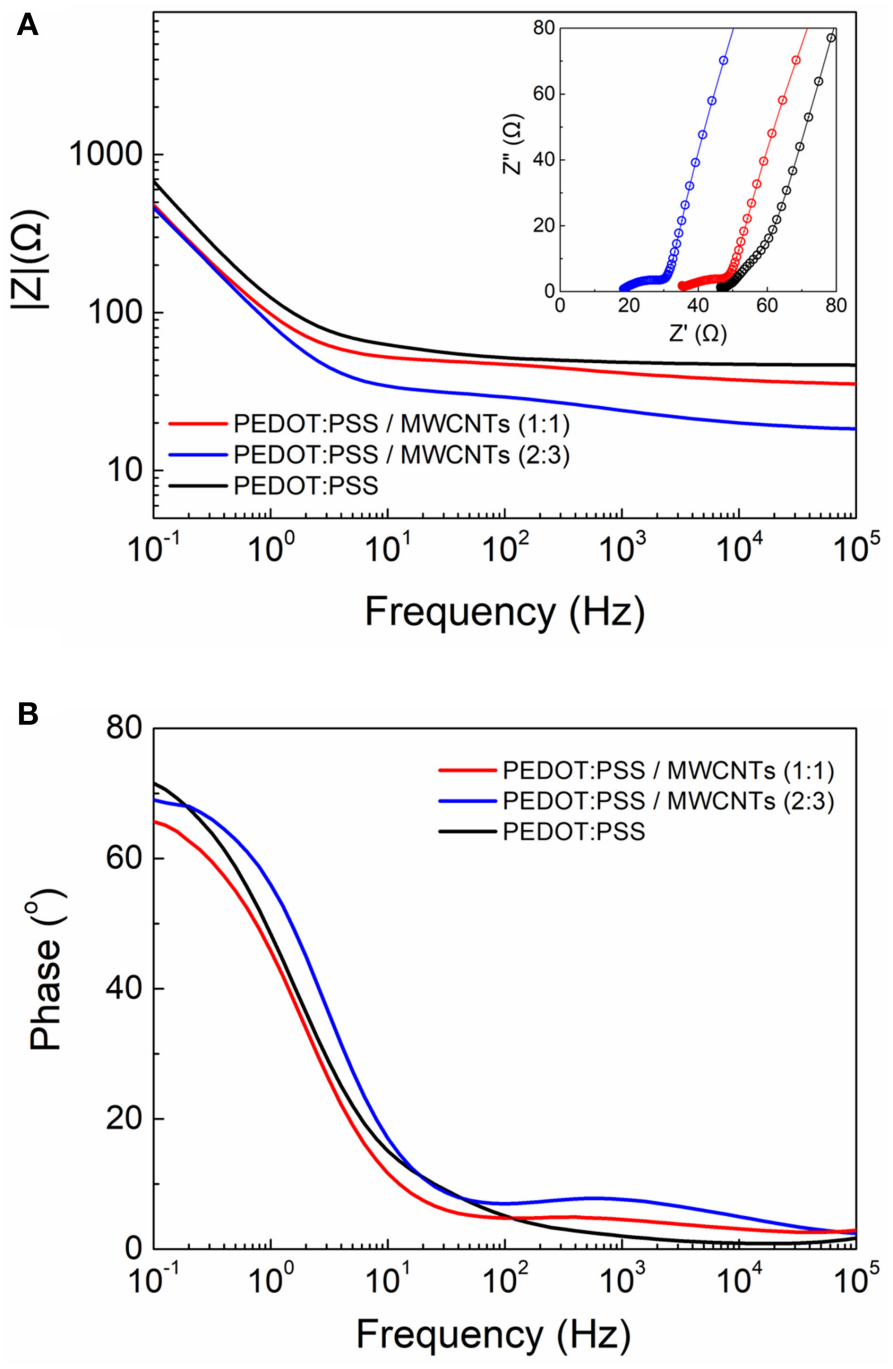
Comparative electrochemical impedance spectroscopy measurements of the PEDOT:PSS and PEDOT:PSS/MWCNT electrodes showing the **(A)** Bode plot (Inset shows the Nyquist plots) and **(B)** the corresponding phase angle diagram.

Furthermore, the scaffolds were electrically characterized by means of electrochemical impedance spectroscopy (EIS). For this set of experiments scaffold-based electrodes were fabricated and measured inside an electrochemical cell. As shown in Figure 4A, the MWCNTs based scaffold electrodes exhibited lower impedance values over the whole frequency spectra when compared to the neat PEDOT:PSS electrodes. At high frequencies the electrodes showed a flat curve characteristic, typically observed for good conducting materials. The apparent differences in the impedance magnitude can be attributed to alterations in the electrical conductivity arising by the inclusion of more electroactive sites in the case of MWCNTs based scaffolds. This effect is more pronounced for the high ratio MWCNTs electrodes. The same trend can be observed at a mid-frequency range (100–1,000 Hz). These observations indicate that the use of MWCNTs have a direct contribution in the enhancement of conductivity in these systems and may offer better sensitivity and operation window for electrical monitoring of biological systems. Moreover, the characteristic “line” observed in the Nyquist plots at the very low frequencies (see inset graphs) is related to ionic diffusion in the bulk of the porous scaffolds. Deviations from “ideal” Warburg diffusion (45° slope) can be attributed to variations in the pore distribution and/or pore geometry within the bulk of the scaffolds (Cooper et al., 2017). Additionally, the MWCNTs based scaffolds exhibited only slight variations compared to pristine, which can be described by a marginal decrease in the phase magnitude (from ~70^o^ to ~65^o^) at low frequencies and a presence of a broad peak at the mid/high frequency range (100–1,000 Hz) (Figure 4B).

We also investigated the use of shorter length MWCNTs (Nanocyl NC7000, 1.5 μm in length) as the size of the CNTs has been shown to play an important role on the overall morphology and electronic properties of CNT-based composites (Russ et al., 2013; Zhou et al., 2015). We thus observed a similar micro-morphology to that of the long MWCNTs-based scaffolds with a nanostructured topography featured by dense ridges at the walls of the pores (Figures S5A,B). Further, we compared the electrochemical properties between the short and the long MWCNTs-based scaffolds by means of EIS (Figure S5C). Interestingly, only a slight increase in the impedance magnitude at the mid/high frequency regime can be observed for the short MWCNTs scaffolds. This can be attributed to the formation of a more extensive conducting network within the long MWCNT-based scaffolds. Nevertheless, short MWCNTs based scaffolds showed overall excellent characteristics, thus represent a cost efficient and very promising alternative to the long MWCNTs based scaffolds.

### Functionalization Capability

**Figure 5:**
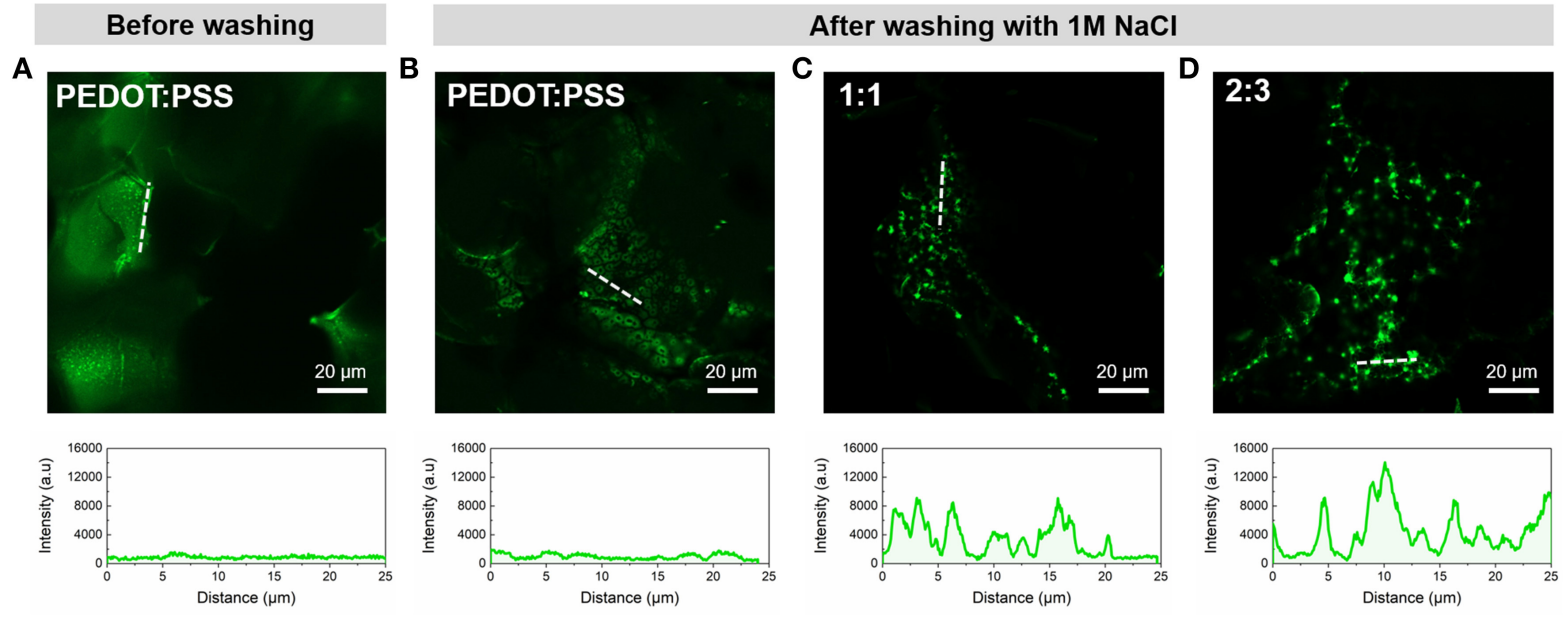
Representative confocal microscopy images of the scaffolds functionalized with PLL-FITC pre- and post-washing and corresponding intensity profiles of regions indicated by white dotted lines; **(A)** PEDOT:PSS scaffold before washing and **(B)** PEDOT:PSS, **(C)** PEDOT:PSS/MWCNT (1:1), and **(D)** PEDOT:PSS/MWCNT (2:3) after-washing with 1M NaCl.

The FITC conjugated bioactive molecule PLL was seen to adsorb on all samples post-incubation, irrespective of whether MWCNTs were present or not. In the case of MWCNT scaffolds this can be explained by the formation of electrostatic interactions between the highly positively charged PLL and the negatively charged oxidized MWCNTs (Ling et al., 2014). However, when the samples were subjected to high ionic strength buffer solution (1M NaCl) in order to remove the excess of PLL-FITC, substantial differences were apparent. The pristine PEDOT:PSS CP scaffold (Figures 5A,B) showed lower fluorescent intensity due to the absence of localized binding, as expected from the non-specific adsorption of PLL on the smooth microstructure. In contrast, Figures 5C,D show significantly higher fluorescent intensities across the PEDOT:PSS/MWCNT hybrid scaffolds in addition to localized “hotspots.” These hotspots can be attributed to the presence of CNT domains in the scaffolds, as evidenced by SEM images and Raman mapping described in section Morphology and Structure. As CNTs have already been demonstrated to improve neurite outgrowth and projection in neural tissue engineering (Hu et al., 2004; Lovat et al., 2005), we assume that the presence of such “hotspots” could possibly provide favorable sites for cell attachment and adhesion, as well as for guiding cell growth (Hirata et al., 2012; Imaninezhad et al., 2018).

### Cytocompatibility

**Figure 6:**
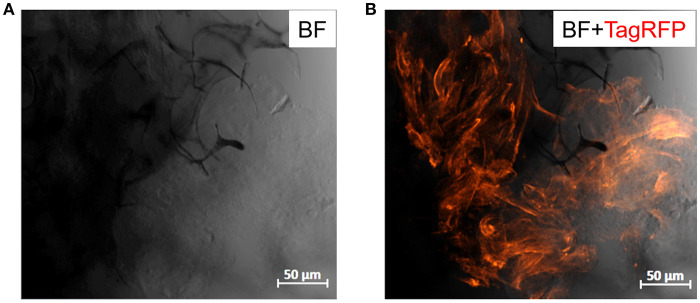
Confocal images of PEDOT:PSS/MWCNTs (2:3) after 2 days in culture with TIF LifeAct cells. **(A)** Bright-field channel illustrating the scaffold porous network (dark gray-black) covered with cells (light gray), **(B)** Bright-field and far-red channels merged highlighting the actin filaments of TIF cytoskeleton (dark orange), as well as the cell growth and penetration into the pores of the scaffold.

The compatibility of PEDOT:PSS with cell cultures is well-established (Ramuz et al., 2015; Ohayon et al., 2017). Several organic electronic schemes, developed by us and others, utilize this material with biological models (e.g., barrier forming cells) for biosensing and monitoring applications (Pappa et al., 2018; Pitsalidis et al., 2018b). Our group has shown that PEDOT:PSS scaffolds are apt structures for generating 3D cell cultures, thanks to their soft, tissue-like nature (Inal et al., 2017). More recently, we developed a 3D bioelectronic device integrated into an electrochemical transistor configuration, that supports the 3D cell culture growth while simultaneously allows for real-time monitoring of the different cell growth stages in a quantitative manner (Pitsalidis et al., 2018a). Herein, we investigated whether the incorporation of the MWCNTs in the PEDOT:PSS scaffolds can affect their cytocompatibility. TIF LifeAct cells (TIFs tagged with RFP labeling the actin filaments in the cytoskeleton), were chosen as a model cell line due to their auto-fluorescence, facilitating faster and more efficient optical monitoring obviating the need for immunofluorescence staining assays, as well as due to their ability to produce extracellular matrix, coating the surface of the scaffolds. The 3D cell culture was maintained in a multi-well plate, where the samples were suspended in complete growth medium. The optical observation (confocal microscopy) of the growth and spreading of the cells over the scaffold was assessed after 2 days. As shown in Figure 6, TIFs appeared to spread over the scaffold, although not in full confluency, presumably because of the short duration of the cell culture. However, the characteristic fibrous shape and individual cell domains of TIFs were formed, in consistence with previous observations (Inal et al., 2017). Although preliminary, these results are promising for engineering PEDOT:PSS/MWCNTs hybrid scaffolds that can serve as hosts of 3D cell culture systems.

## Conclusions

CP scaffolds have come to the fore as multifunctional smart biomaterials acting simultaneously as templates for 3D tissue growth as well as recording or stimulating electrodes for the growing tissue. Such dual capabilities have great implications in tissue engineering both *in vitro* and *in vivo*. Likewise, 3D electrodes that are structurally and mechanically compliant with tissue can also have significant implications in next generation medical prosthesis and bioelectronic therapies. In previous studies we have highlighted the potential of PEDOT:PSS as an excellent candidate material for the formation of 3D porous biomimetic structures/electrodes promoting tissue growth in 3D and allowing for quantitative assessment of tissue formation to be elucidated through deconvolution of the electrical measurements. In this work, we have performed a systematic study on the effect of MWCNTs addition on the electronic and morphological properties of PEDOT:PSS scaffolds. Alongside the enhancement in the electrical conductivity, the MWCNT-modified scaffolds exhibited good cytocompatibility and biofunctionalization (i.e., PLL) capability, important for biosensing and cell attachment (i.e., to promote adhesion of specific cell types). Overall, such high-performance systems can be useful for applications where electronic performance is particularly important, such as in applications for tissue stimulation and electronic implants.

## Author Contributions

CP and AJ contributed equally to this work. CP and AJ developed the scaffolds and perform electrical characterization. AJ performed the morphological characterization and the functionalization of the scaffolds. Cell experiments and confocal measurements were carried out by C-MM. ET characterized the films using Raman spectroscopy. CP conceived the idea. RO, CP, MD, and J-SK supervised the research work and reviewed the results. CP and AJ wrote the paper.

### Conflict of Interest Statement

The authors declare that the research was conducted in the absence of any commercial or financial relationships that could be construed as a potential conflict of interest.
