# Corrigendum: 3D Hybrid Scaffolds Based on PEDOT:PSS/MWCNT Composites

**DOI:** 10.3389/fchem.2020.00698

**Published:** 2020-08-07

**Authors:** Akhila K. Jayaram, Charalampos Pitsalidis, Ellasia Tan, Chrysanthi-Maria Moysidou, Michael F. L. De Volder, Ji-Seon Kim, Roisin M. Owens

**Affiliations:** ^1^Department of Chemical Engineering and Biotechnology, University of Cambridge, Cambridge, United Kingdom; ^2^Department of Physics and Centre for Plastic Electronics, Imperial College London, London, United Kingdom; ^3^Department of Engineering, University of Cambridge, Cambridge, United Kingdom

**Keywords:** carbon nanotubes, conducting scaffolds, porous, PEDOT:PSS, electrode

In the original article, we neglected to include the funder European Research Council (ERC) under the European Union's Horizon 2020 research and innovation programme, grant agreement No. 723951 to RO. The corrected Funding section reads as follows:

This work was supported by the EPSRC Cambridge NanoDTC, EP/L015978/1 and the European Research Council (ERC) under the European Union's Horizon 2020 research and innovation programme, Grant Agreement No. 723951 to RO.

The authors apologize for this error and state that this does not change the scientific conclusions of the article in any way. The original article has been updated.

