# Supplementary material for: 3D Hybrid Scaffolds Based on PEDOT:PSS/MWCNT Composites
Source: Front Chem. 2019 May 21;7:363. doi: 10.3389/fchem.2019.00363 (PMC6536663; doi:10.3389/fchem.2019.00363)
Supplement: Supplementary file 1 [file Table_1.DOCX]

Supplementary Material

# Supplementary Figures


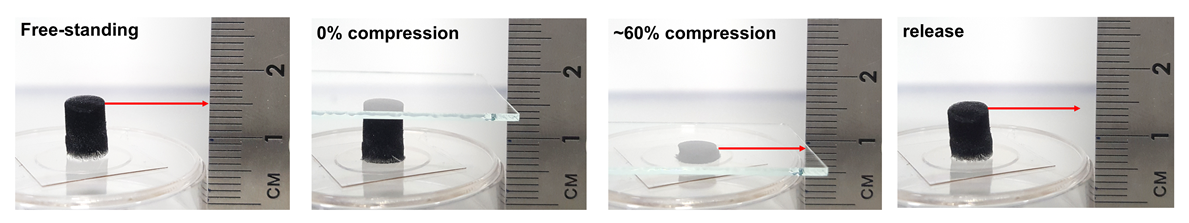


**Figure S1.** Qualitative compression test on a PEDOT:PSS/MWCNT (2:3 ratio) scaffold where a unidirectional compressive load was exerted. Illustrated photos showing a complete compression test cycle. The scaffold was found to recover its initial shape after compression.

**
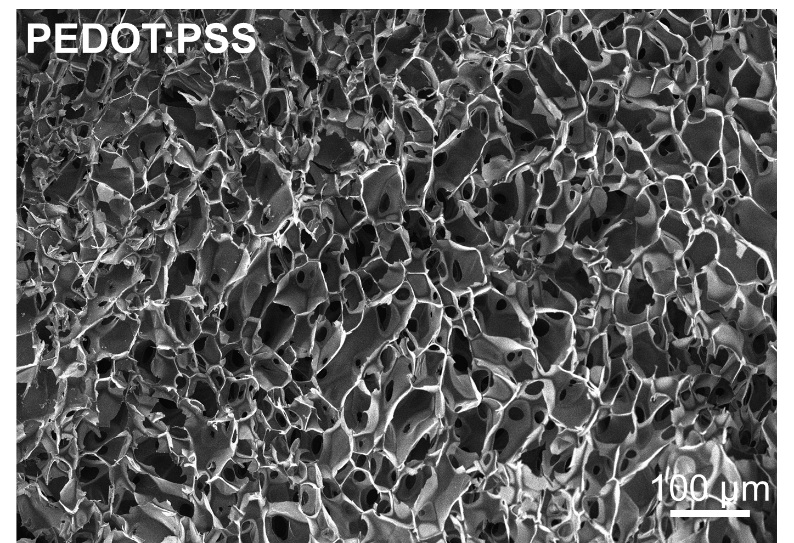
**

**Figure S2.** SEM image showing the porous morphology of a pristine PEDOT:PSS scaffold.

**
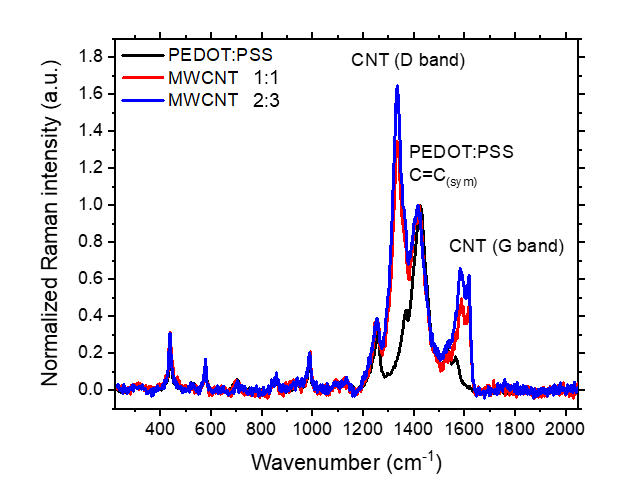
**

**Figure S3.** Raman spectra acquired in the 2D Raman mapping scans of the reference PEDOT:PSS and the PEDOT:PSS/MWCNT (1:1 and 2:3) scaffolds. These spectra were used to construct the overall 2D Raman map.

**
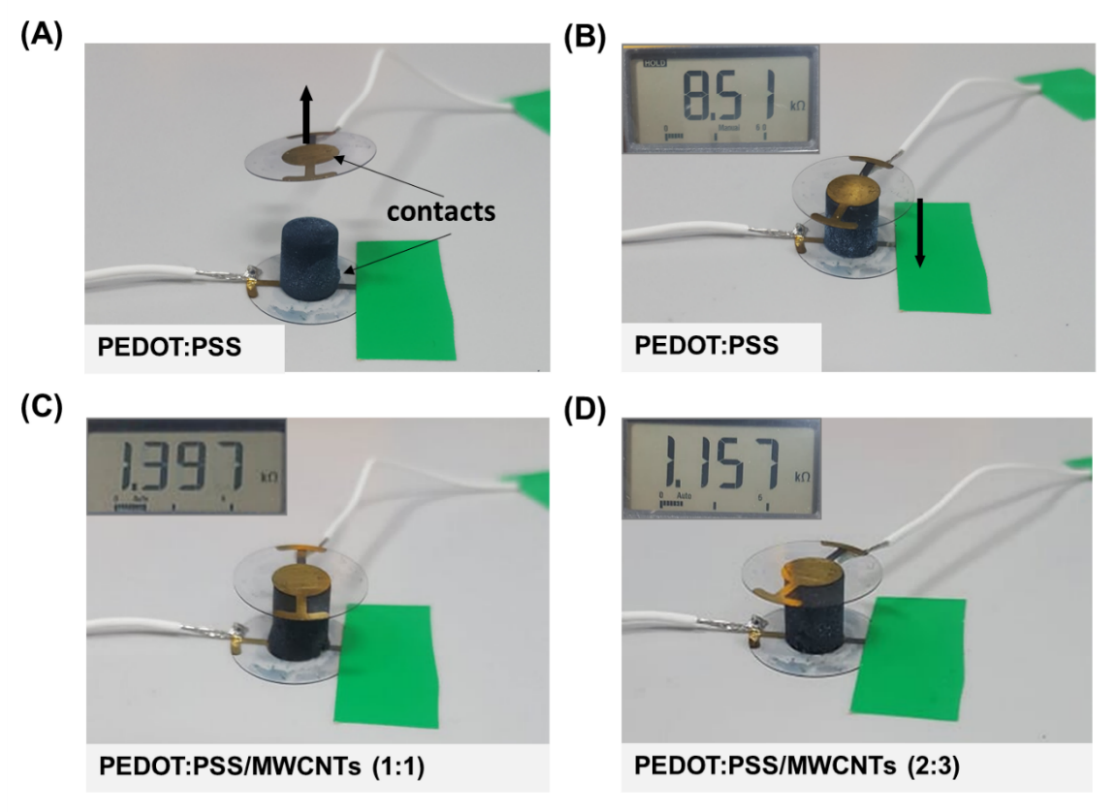
**

**Figure S4.** Measurement of electrical resistance between two contact points in the various dry scaffolds; **(A)** Basic design of experimental setup and measurement of the electrical resistance of **(B)** pristine PEDOT:PSS scaffold, PEDOT:PSS/MWCNT scaffolds with **(C)** 1:1 ratio and **(D)** 2:3 ratio. Insets show the multimeter values of the resistance for each scaffold.

**
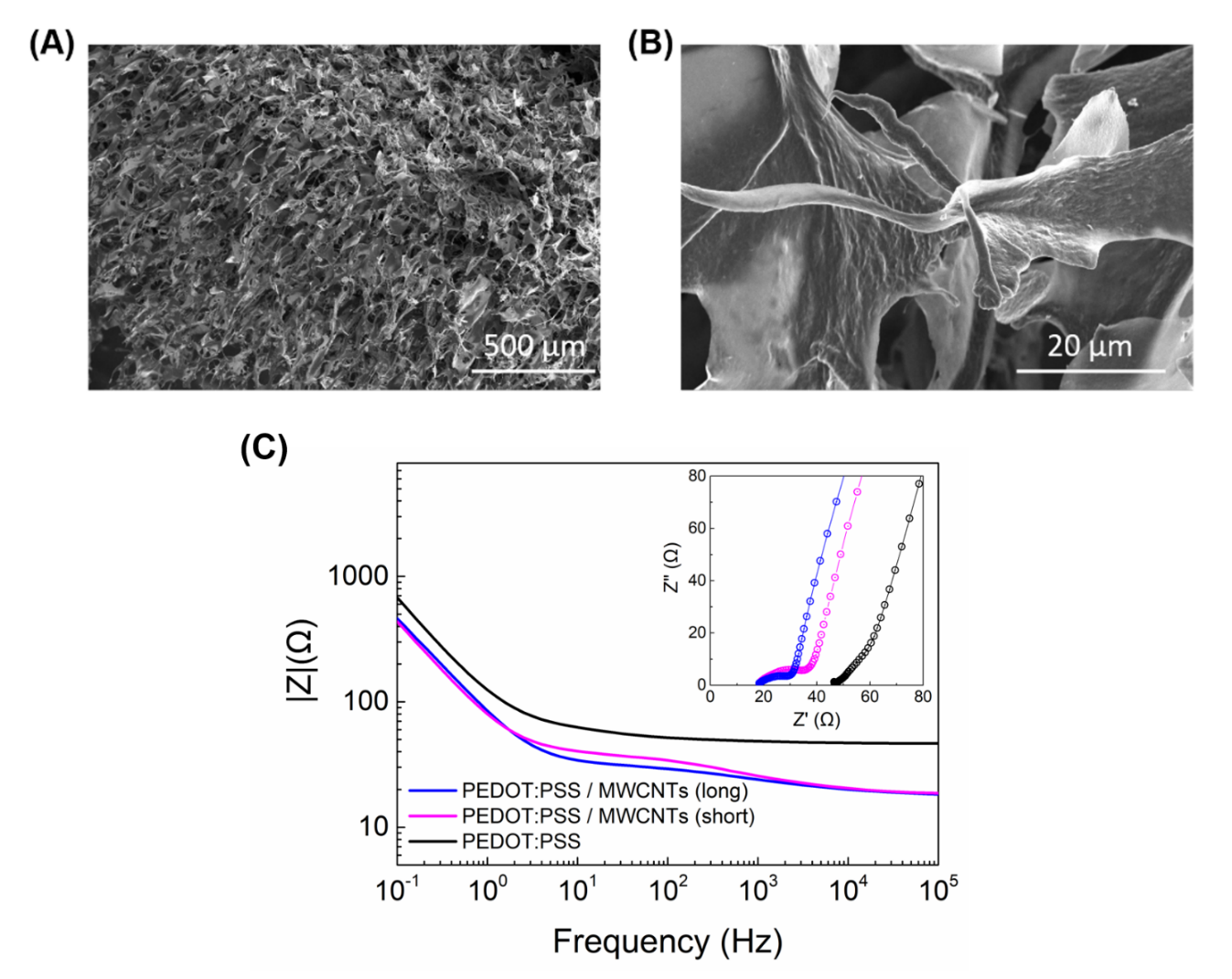
**

**Figure S5.** SEM Images of the PEDOT:PSS/short-MWCNTs (at ratio 2:3) scaffold at (**A**) low and (**B**) high magnification. (**C**) Comparative electrochemical impedance spectroscopy measurements (Bode plot) of the PEDOT:PSS/long-MWCNTs, PEDOT:PSS/ short-MWCNTs and pristine PEDOT:PSS scaffolds. Inset graph shows the corresponding Nyquist plot.

# Supplementary Data

**Synthesis of Oxidised MWCNTs**

MWCNTs from Microphase (LLCNTs, 300 μm in length) were oxidised using a microwave (Anton Paar MultiwavePro). The CNTs were dispersed in 20 mL of nitric acid (Fisher Scientific) each, heated to 180°C in a 10-minute ramp and maintained at 180°C for 30 minutes using IR-control. The resulting solution was washed with deionised water to increase the pH and vacuum filtered until a neutral pH was achieved. The powder was transferred to a vial with ethanol and dried overnight in an oven at approximately 70°C. The dried powder was redispersed in MilliQ water to obtain a 0.1 wt% solution.
